# Analysis of the Ion Adsorption–Desorption Characteristics of Biofilm Matrices

**DOI:** 10.1264/jsme2.ME11339

**Published:** 2012-05-17

**Authors:** Andi Kurniawan, Tatsuya Yamamoto, Yuki Tsuchiya, Hisao Morisaki

**Affiliations:** 1 Graduate School of Science and Engineering, Ritsumeikan University, 1–1–1 Noji Higashi, Kusatsu, Shiga, 525–8577, Japan

**Keywords:** biofilm, biofilm polymer, ion adsorption-desorption characteristics, nutrient ions

## Abstract

The characteristics of biofilm polymers formed on stone surfaces in Lake Biwa and ion adsorption and desorption to and from these biofilms were investigated. The results indicated that both positively and negatively charged sites exist in the biofilm polymer. A physicochemical interaction between these sites and ions in the surrounding water seems to promote the adsorption of ions to the biofilm through an attractive electrostatic interaction and an ion-exchange mechanism. The results also indicated that, in comparison with ion-exchange resins, ions were more loosely bound to and desorbed more easily from the biofilm polymer. This suggests that microbes in the biofilm can readily use these ions as nutrient ions. Our present findings indicate that the biofilm may play an important role in supplying nutrient ions to microbes in the biofilm and in the development of a nutrient-rich environment within the biofilm through both ion adsorption and desorption. This study shows for the first time that the inside of a biofilm can be a sustainable environment for microbes.

A large variety of microbes live in abundance in biofilms and form community structures different from those in the surrounding environments (2, 18, 20, 27, 31). The community structure specific to biofilms seems to be the result of the different environments inside and outside the biofilm.

Nutrient-ion concentrations within the biofilm have been shown to be one hundred- or thousand-fold higher than those in the surrounding lake water (26). These high nutrient-ion concentrations in the biofilm seem to be an important factor that affects microbial growth and survival in the biofilm and result in a microbial community structure that is different from that of the external environment.

In the previous study, we proposed that the adsorption of nutrient ions from the surroundings to the biofilm formed a nutrient-rich environment within the biofilm (13, 26). In the present study, we aimed to investigate ion adsorption and desorption on a biofilm formed in a natural environment in detail. Specifically, by comparing the biofilm with ion-exchange resins, we studied ion-adsorption kinetics, the adsorption isotherms of various ions, and ion desorption, which possibly occurs by ion exchange with the added ions. Such detailed investigations have not been performed in recent studies, which focused mainly on the removal of pollutants such as heavy metal ions by using biofilms (7, 23–25).

In the present study, we showed that the main driving force for ion adsorption to the biofilm matrix is a physicochemical interaction between the charged sites in the biofilm polymer and the ions in the surrounding water. Analysis of the ion-adsorption isotherms for the biofilm revealed that adsorbed ions can be more loosely bound to and desorbed more easily from the biofilm polymer than ion-exchange resins. The biofilm seems to form a stable and sustainable environment for microbes through the adsorption of ions from the external environment to supply the microbes inside.

## Materials and Methods

### Sampling site and sample preparation

In this study, we used biofilms formed on the surface of stones on the shore of the southern basin (Akanoiwan) of Lake Biwa, Japan. The stones were collected at the end of December 2008 and in early January 2010 (by using electrophoretic mobility [EPM] measurements in our preliminary research, we confirmed that the biofilms collected in December and early January had a similar electric charge).

The stones (ca. 100 stones on each sampling day) were collected from a depth of ca. 50 cm in the lake and were brought back to the laboratory in a plastic container filled with lake water from the same site. The temperature of the container was maintained at 4°C.

The biofilms were removed from the surfaces of the stones with a sterilized toothbrush and then suspended in distilled water. The suspensions were centrifuged (8,000×*g* at 4°C for 10 min), and biofilm pellets were obtained. All pellets were stored at −40°C until their use in the experiments.

The pellet of the biofilms collected in December 2008 was divided into 3 parts. The first part was washed 6 times with 5 mM phosphate buffer (pH 7) with repeated centrifugation. The obtained biofilm was resuspended in 5 mM phosphate buffer (pH 7), and the suspension was used to examine the kinetics of cation adsorption. The second part of the pellet was washed 6 times with distilled water with repeated centrifugation and then resuspended in distilled water. The suspension was used for potentiometric titration and to examine the kinetics of anion adsorption. Distilled water was used instead of phosphate buffer to avoid the influence of phosphate buffer on the potentiometric titration and the influence of PO_4_^3−^ (one of the anions considered) contained in the buffer on the anion-adsorption experiment. The last part of the pellet was washed 3 times with 10 mM NaCl solution with repeated centrifugation and resuspended in 1 mL of 10 mM NaCl solution. The suspension was mixed vigorously and dispersed by vortexing (Vortex Genie 2; M&S Instrument, Osaka, Japan; 3,000 rpm) for 5 min and sonication (2510J-MT; Yamato Scientific, Tokyo, Japan; 42 kHz, 125 W) for 10 min, followed by vortexing for 30 s. The resulting biofilm suspension was used to analyze the electric charge of the biofilm.

The pellet of the biofilms collected on January 2010 was divided into 2 parts. The first part was washed 6 times with phosphate buffer (pH 7) with repeated centrifugation and then resuspended in phosphate buffer (pH 7). The suspension was used for the analysis of cation-adsorption isotherms. The other part of the pellet was washed 6 times with distilled water with repeated centrifugation and then resuspended in distilled water. This suspension was used for the analysis of anion-adsorption isotherms.

### Dry weight of the biofilm

The biofilm pellet (ca. 1 wet-g) was dehydrated for 3 d at 65°C until a stable weight was obtained.

### Electric charge of the biofilm at various pH values

One milliliter of the biofilm suspension (containing ca. 0.03 wet-g biofilm) was placed in an electric field, and the electrophoretic mobility (EPM) of the dispersed biofilm was measured with a ZETASIZER Nano-Z (Malvern Instruments, Worcestershire, UK) at pH 2–9 in 10 mM ionic strength phosphate-buffered saline (PBS), as described in detail previously (13).

### Potentiometric titration of the biofilm

Using an automatic titrator (DL50; Mettler Toledo, Columbus, OH, USA), 100 mM HCl or NaOH solutions were titrated into 40 g biofilm suspension (containing 1 wet-g biofilm), and the changes in pH were analyzed. Lake water (40 mL) and distilled water (40 mL) were also used for comparison. From the results, the uptake capacities of H^+^ or OH^−^ by the biofilm and lake water were calculated as follows:

(1) $$\text{C}({\text{H}}^{+})=\frac{(10^{-\text{pH}(\text{dw})}-10^{-\text{pH}(\text{sample})})v}{w}$$

where C(H^+^) is the uptake capacity of a proton (H^+^) at a given pH per unit weight of biofilm (μmol wet-g^−1^) or lake water (μmol g^−1^), pH (dw) and pH (sample) are the pH values of distilled water and the sample (biofilm suspension or lake water), respectively, when the same amount of HCl or NaOH solution was added. The term *v* represents the sample volume (L), and *w* is the wet weight of the biofilm (wet-g) or the weight of lake water (g).

When NaOH solution was added, the uptake capacity of hydroxide ions (OH^−^) per unit weight of biofilm or lake water, C(OH^−^) was obtained by a similar equation as follows:

(2) $$\text{C}({\text{OH}}^{-})=\frac{(10^{-\text{pH}(\text{sample})}-10^{-\text{pH}(\text{dw})})v}{w}$$

where C(OH^−^) is the uptake capacity of OH^−^ ions by the biofilm or lake water at a given pH. The other terms are the same as above.

### Kinetics of ion adsorption and desorption

In this study, we examined the ions (NH_4_^+^, K^+^, Ca^2+^, Mg^2+^, NO_3_^−^, and Cl^−^) commonly present in the interstitial water of biofilms formed in Lake Biwa, as confirmed in the preliminary research.

Five milliliters of 20 mM NH_4_Cl, KCl, CaCl_2_, or MgCl_2_ aqueous solutions (for cation adsorption) and NaCl or NaNO_3_ aqueous solutions (for anion adsorption), prepared by diluting the reagent grade chemical compound in 5 mM phosphate buffer (pH 7) or distilled water, were added to 45 mL samples of the biofilm suspension (containing 1 wet-g biofilm). The suspension was maintained at 25°C by using a thermostatic water bath and mixed well with a magnetic stirrer (ca. 700 rpm).

Aliquots of the suspension were subsampled at various time intervals (0.5, 1, 3, 5, 10, 20, 30, and 60 min after adding the test solution for cation or anion adsorption) and then centrifuged (15,000×*g* at 4°C for 1 min) to obtain the supernatant. The ion concentration in the supernatant was measured by capillary electrophoresis (CAPI-3300; Otsuka Electronics, Osaka, Japan).

Fifty milliliters of 5 mM phosphate buffer (pH 7) and distilled water (without the biofilm) were used as the controls for the cation-adsorption and anion-adsorption experiments, respectively. The quantity of ions adsorbed to the biofilm was calculated from the difference between the ion concentrations in the subsamples and the control.

Other cations, different from those adsorbed, that desorbed from the biofilm by ion exchange were also investigated. The amounts of desorbed cations were calculated from the differences between the measured concentrations of the desorbed cations, after the addition of the ion under consideration, and the background concentrations. To determine the background concentrations, the same amount of biofilm was resuspended in 50 mL of 5 mM PBS (pH 7), followed by the same procedure as described above without ion addition.

The adsorption kinetics and desorption experiment were also conducted for 1 dry-g of a strongly (SP-650M; Toyopearl, Tokyo, Japan) and weakly (CM-650M; Toyopearl) acidic (cation exchange) resin, a strongly (SuperQ-650M; Toyopearl) and weakly (DEAE- 650M; Toyopearl) basic (anion exchange) resin, and an electrically neutral resin (HW-65; Toyopearl).

### Adsorption isotherm

Two milliliters of 3 mM NH_4_Cl or MgCl_2_ aqueous solutions (for cation adsorption) and NaNO_3_ or Na_3_PO_4_ aqueous solutions (for anion adsorption), prepared using the same method as described above, was added to 8 mL samples of the biofilm suspension (containing 0.5 wet-g biofilm) and mixed well using a magnetic stirrer (ca. 700 rpm). After 5 min, 3 mL of the suspension was subsampled and centrifuged (8,000×*g* at 4°C for 3 min) to separate the centrifuged pellet and the supernatant. Two milliliters of the obtained supernatant was used to measure the ion concentration using capillary electrophoresis (CAPI-3300; Otsuka Electronics), while the remaining 1 mL of the subsample (leftover supernatant and centrifuged pellet) was resuspended in the original sample suspension.

To increase the ion concentration stepwise, we repeated the above procedure 6 times using a 3-mM concentration of the ion solution, then 6 times using a 20-mM concentration, and finally 3 times using a 100-mM concentration.

The amount of ions adsorbed to the biofilm was calculated from the difference between the amount of ions added to the biofilm suspension and the actual amount of ions in the biofilm suspension after each addition of ion solution.

The maximum adsorption amount and the adsorption equilibrium constant for ion adsorption were calculated using a variant of the Langmuir isotherm equation (3, 10, 19, 28), as described below.

(3) $$\frac{C}{N}=\frac{1}{(N_{\text{max}})b}+\frac{C}{N_{\text{max}}}$$

This equation is based on the assumption that a dynamic equilibrium exists between the adsorbed ion (*N*; mmol dry-g^−1^) and the free ion in solution, the concentration of which is given as an equilibrium concentration (*C*; mM). The adsorption equilibrium constant (*b*) was defined as the ratio of the adsorption and desorption rates. The value of *b* increases as the adsorption rate exceeds the desorption rate, relatively. The plot of *C/N* against *C* yields a straight line with a slope of 1/*N*_max_ and a *y*-axis intercept of 1/(*N*_max_)*b*, and thus, the values of *N*_max_ (the maximum amount of adsorbed ion; mmol dry-g^−1^) and *b* can be calculated (10).

To prevent ion uptake by microbes in the biofilm, all the adsorption isotherm experiments were conducted in an ice bath (ca. 0°C).

The adsorption isotherm experiments were also conducted for 0.5 dry-g of the same types of resins as those used in the adsorption kinetics experiment.

## Results and Discussion

### Electric charge of the biofilm

**Fig. 1:**
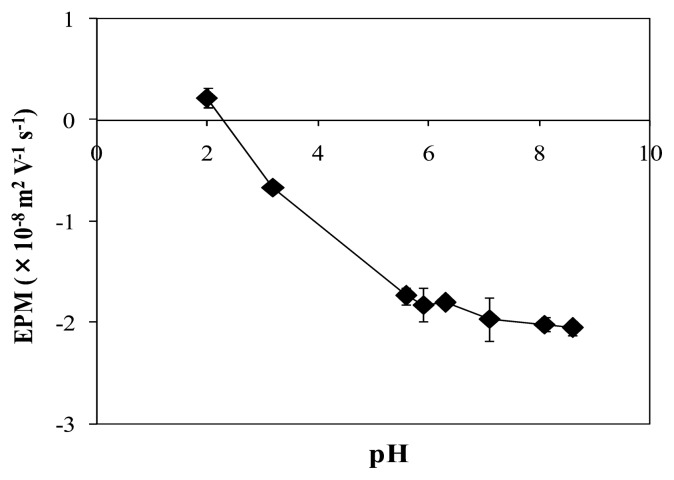
Change in the electrophoretic mobility (EPM) of the biofilm with pH. EPM was measured under various pH conditions of 10 mM ionic strength. Bars represent the standard error.

The biofilm showed a negative EPM at pH 7, and the value gradually decreased with decreasing pH, especially at around pH 4 (Fig. 1). This was probably due to the acidic environment that suppressed the ionization of negatively charged functional groups in the biofilm polymer, such as the carboxylic group whose p*K*a is around pH 4.

The biofilm exhibited a positive EPM at pH 2, indicating the existence of positively charged functional groups, thought to be amino groups (to be discussed later), in the biofilm polymer.

The pH of the buffer solution used in the EPM measurement increased after the biofilm samples were added, *e.g.*, buffer solutions with pH 4.0 and 5.0 shifted to 5.6 and 5.9, respectively. This indicated a decrease in the concentration of protons by addition of the biofilm samples. Protons seem to de-ionize acidic functional groups such as the carboxylic group, which is the most probable acidic functional group in the biofilm polymer.

The relatively large standard error values at pH 6 and pH 7 may be attributable to the heterogeneity of the biofilm components (4). In this study, since several samples of varying pH (pH 2.0 to pH 9.0) were required to measure the EPM, the biofilm suspension was divided into several parts. Hence, the components of each biofilm sample used in the EPM measurement may not be identical.

### Potentiometric titration of the biofilm

**Fig. 2:**
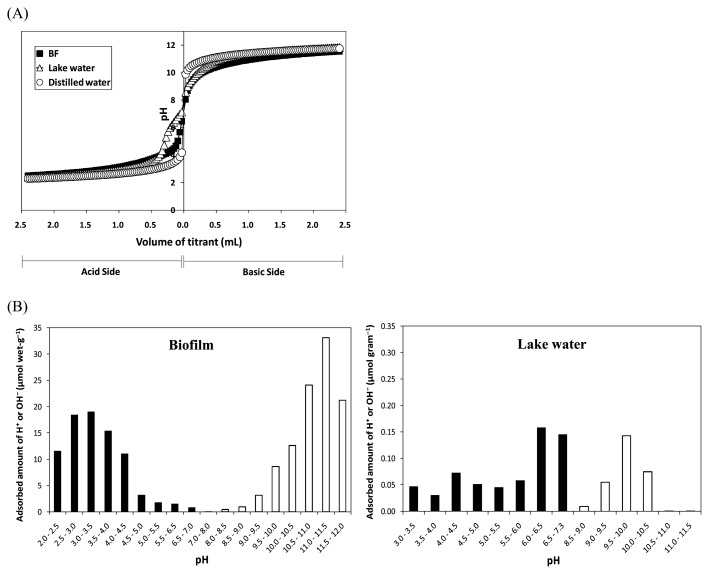
(A) Potentiometric titration of the biofilm, lake water, and distilled water, and (B) the adsorbed amount of H^+^ (black column) or OH^−^ (white column) per unit weight of biofilm and lake water, calculated using equations 1 and 2 described in Materials and Methods.

Potentiometric titrations of the biofilm suspension, lake water, and distilled water were performed (Fig. 2A). From the results shown in Fig. 2A, the uptake capacities of H^+^ and OH^−^ by the biofilm and by lake water were calculated (Fig. 2B). The biofilm showed greater uptake capacity at around pH 4 for H^+^ (ca. 19 μmol wet-g^−1^) and at around pH 11 for OH^−^ (ca. 32 μmol wet-g^−1^) (Fig. 2B). Lake water also had a maximum uptake capacity, albeit small, at around pH 4 and 6 for H^+^ (ca. 0.08 μmol g^−1^ and ca. 0.14 μmol g^−1^, respectively) and at around pH 10 for OH^−^ (ca. 0.14 μmol g^−1^).

The large uptake capacity of the biofilm at around pH 4 seems to be attributable to the protonation of a functional group such as the carboxylic group, whose p*K*a is ca. pH 4, in the biofilm polymer. Another high capacity value for the biofilm at around pH 11 seems to be attributable to the neutralization of OH^−^ with H^+^, released by the de-protonation of functional groups such as the amino group, whose p*K*b is ca. pH 11. Thus, these results indicate the existence of functional groups such as carboxylic and amino groups in the biofilm polymers. The uptake capacity of lake water may indicate that some substances carrying ionizable functional groups already existed in the lake water. Suspended organic solids such as humic substances, which possess uptake capacities for H^+^ or OH^−^, may be present in the lake water (16).

The existence of carboxylic and amino groups in biofilm polymers provides an explanation for the EPM change in the biofilm with a change in pH, as shown in Fig. 1. At pH 7, the carboxylic group is ionized to bear a negative charge and the amino group is protonated to carry a positive charge. In the case of a greater amount of negative charge than positive charge in a sample, the sample will have a net negative charge. The negative EPM of the biofilm at pH 7 seems to correspond to this case. Thus, the biofilm polymer seems to carry both positive and negative charges in the environment of lake water whose pH is ca. 7. This leads to the assumption that the biofilm can interact with both cations and anions in the lake water via electrostatic interaction.

### Kinetics of ion adsorption and desorption

**Fig. 3:**
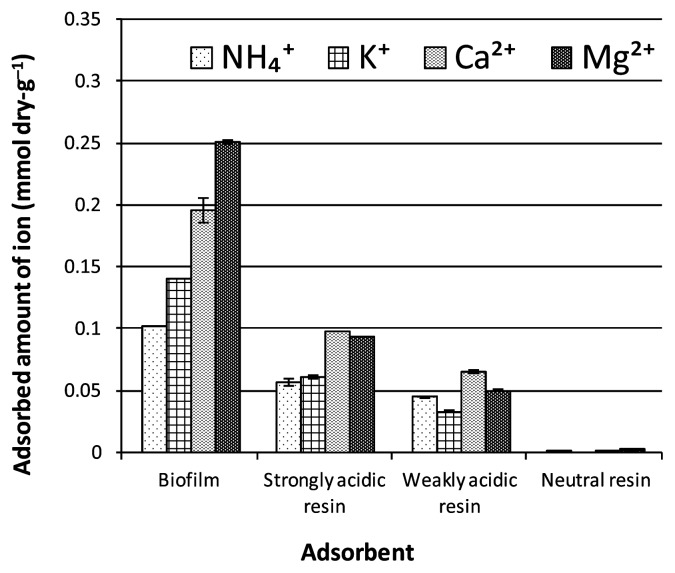
The amount of various cations adsorbed to the biofilm and acidic resins.

The time course of cation adsorption to the biofilm was investigated by comparison with cation-exchange resins (strongly acidic and weakly acidic resins) (data not shown). All the cations examined (NH_4_^+^, K^+^, Ca^2+^, and Mg^2+^) were adsorbed to the biofilm in quite a short time. Within 1 min, the adsorption amount attained was not exceeded for the rest of the experiment, and this was also the case for the cation-exchange resins. Divalent cations (Mg^2+^ and Ca^2+^) were adsorbed to a greater extent than monovalent cations (K^+^ and NH_4_^+^) to both the biofilm and the ion-exchange resins (Fig. 3); however, none of the cations were adsorbed to the electrically neutral resin (Fig. 3). Thus, cation adsorption on the biofilm seems to occur as a physicochemical process, where the electrostatic force between the cations and the negatively charged sites in the biofilm polymer is the driving force.

**Fig. 4:**
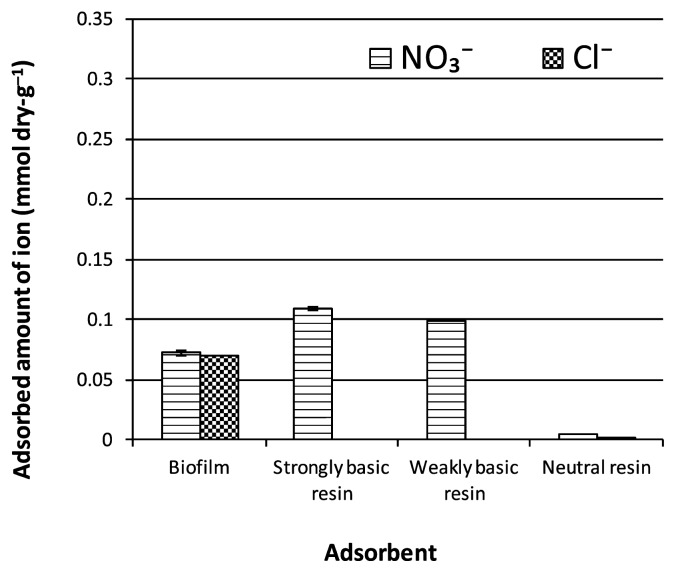
The amount of anions adsorbed to the biofilm and basic resins.

Anion adsorption to the biofilm and anion-exchange resins (strongly basic and weakly basic resins) was also investigated (Fig. 4). The biofilm adsorbed nitrate anions as fast as the anion-exchange resins, and the amount of adsorption observed within 1 min was not exceeded for the rest of the experiment (data not shown). Chloride and nitrate anions, which have the same valency (the same amount of charge), were adsorbed to the biofilm in similar magnitudes (0.070 mmol dry-g^−1^ and 0.075 mmol dry-g^−1^, respectively) (Fig. 4). Conversely, electrically neutral resins could not adsorb anions (Fig. 4).

There was a possibility that the adsorption of ions to the biofilm was not only caused by the physicochemical interaction between the ions and the biofilm polymer but also by active uptake by viable microbes in the biofilm. To clarify this, a biofilm sample was autoclaved and used as an adsorbent. The adsorbed amount of cations to the autoclaved biofilm (0.12 mmol dry-g^−1^ and 0.23 mmol dry-g^−1^ for K^+^ and Mg^2+^, respectively) was slightly less than that to the intact biofilm (0.14 mmol dry-g^−1^ and 0.25 mmol dry-g^−1^ for K^+^ and Mg^2+^, respectively). This indicated that the adsorption of ions was caused primarily by a nonbiotic process. The EPM value of the autoclaved biofilm was almost the same as that of the intact biofilm (average difference, ca. 0.03%). This indicated that autoclaving had no significant effect on the electric charge of the biofilms.

The ion adsorption rate of the biofilm was compared to the ion consumption rate of the bacterial cells. The K^+^ ion consumption rate of *Escherichia coli* was reported to be ca. 6×10^−16^ mmol cell^−1^ min^−1^ (17). Assuming that all cells in the biofilm have the same ion consumption activity as *E. coli*, the K^+^ ion consumption rate of the bacterial cells can be estimated to be ca. 6×10^−7^ mmol min^−1^ for 1 wet-g biofilm, which contains ca. 10^9^ bacterial cells on average (13). This value is far smaller than the ion adsorption rate of biofilms found in this study, *i.e.*, for K^+^, ca. 3×10^−2^ mmol (wet-g biofilm)^−1^ min^−1^. This indicates that ion adsorption to the biofilm has greater ability to take up ions from the surrounding water than ion consumption by bacterial cells in an aquatic environment. However, this estimate was made using the results of laboratory-scale experiments, and it must be noted that these conditions might be different from actual environmental conditions.

During the adsorption of cations to the biofilm or cation-exchange resins, other types of cations (different from the adsorbing one) may be released into the solution. Through an ion-exchange mechanism, the adsorbing cations seem to promote the desorption of other cations that were previously bound to negatively charged sites in the biofilm.

In the case of ion-exchange resins as the adsorbent, the total amount of desorbed cations, *i.e.*, Na^+^ in mmol dry-g^−1^, was almost equal to that of adsorbed cations for monovalent cations (NH_4_^+^ and K^+^) as the adsorbate and about twice that for divalent cations (Ca^2+^ and Mg^2+^) (Table 1). In the ion-exchange mechanism, every ion adsorbed from the solution to the adsorbent replaces an equivalent amount of charge of a previously adsorbed similarly charged exchangeable ion. Thus, when only monovalent cations are desorbed from the adsorbent, the amount of adsorbed cations is approximately equal to that of desorbed cations for monovalent cations as the adsorbate and half of that for divalent cations. This was the case for ion-exchange resins.

In the case of a biofilm as the adsorbent, both monovalent (NH_4_^+^, K^+^, and Na^+^) and divalent (Ca^2+^ and Mg^2+^) cations were desorbed during ion adsorption (Table 1). In terms of the amount of charge (calculated from the amount of cations [Table 1]), the desorbed amount of cations from the biofilm (0.051 meq dry-g^−1^, 0.11 meq dry-g^−1^, 0.061 meq dry-g^−1^, and 0.13 meq dry-g^−1^ for NH_4_^+^, K^+^, Ca^2+^, and Mg^2+^ as the adsorbate, respectively) was lower than the adsorbed amount of cations (0.103 meq dry-g^−1^, 0.14 meq dry-g^−1^, 0.41 meq dry-g^−1^, and 0.50 meq dry-g^−1^ for NH_4_^+^, K^+^, Ca^2+^, and Mg^2+^ as the adsorbate, respectively); note that all cations in the solution were identified using a capillary method (*i.e.*, there was no unidentified ion in the solution). This indicated that the cations from the surrounding water not only replaced the previously bound cations on the biofilm polymer through the ion-exchange mechanism but were also retained in the biofilm matrix through an attractive electrostatic interaction with negative charges in the biofilm polymer. At least, the amount of cations desorbed may indicate the contribution of the ion-exchange mechanism to ion adsorption to the biofilm. On the other hand, the differences between the amount of cations adsorbed and desorbed, in terms of the amount of charge, may be considered as the contribution of the attractive electrostatic interaction to ion adsorption to the biofilm. The contribution of the attractive electrostatic interaction in the case of divalent cations as the adsorbate (0.35 meq dry-g^−1^ and 0.38 meq dry-g^−1^ for Ca^2+^ and Mg^2+^ as the adsorbate, respectively) was greater than that of monovalent cations as the adsorbate (0.052 meq dry-g^−1^ and 0.030 meq dry-g^−1^ for NH_4_^+^ and K^+^ as the adsorbate, respectively). This indicates that the attractive electrostatic force between the biofilm and divalent cations is stronger than that between the biofilm and monovalent cations.

### Adsorption isotherm

In order to examine in more detail the characteristics of ion adsorption-desorption in the biofilm, the adsorption isotherms of various ions to the biofilm were studied.

**Fig. 5:**
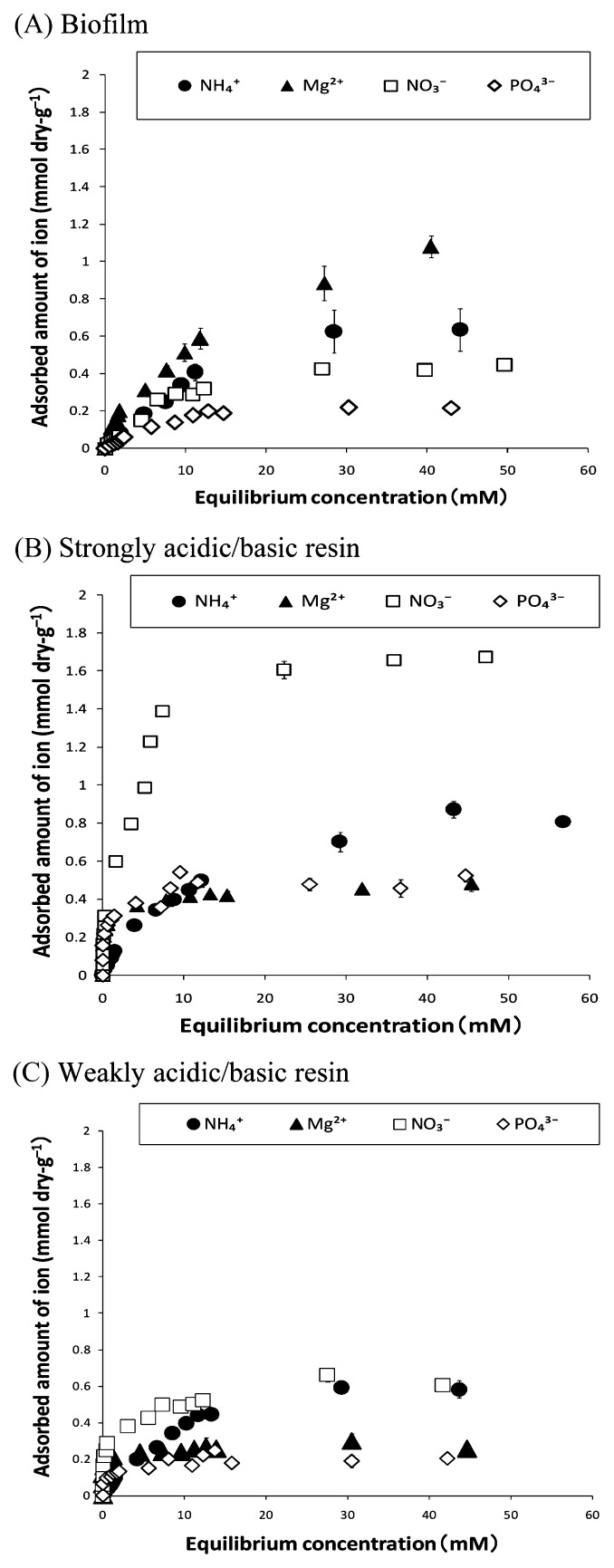
Adsorption isotherm for various ions on (A) biofilm, (B) strongly acidic or basic resins, and (C) weakly acidic or basic resins. The adsorbates were different anions or cations (see the inset). For adsorption of various ions on biofilm (A), open marks (□, ⋄) and closed marks (●, ▲) represent anion and cation adsorption on the biofilm, respectively. In the case of ion-exchange resins (B and C), open marks (□, ⋄) represent anion adsorption by basic resins, and closed marks (●, ▲) represent cation adsorption by acidic resins. Owing to limitations of the sample, the experiments on the adsorption of anions on the biofilm were conducted only once. Other experiments were repeated 2 to 3 times, independently (average values are shown). Bars represent the standard error.

As shown in Fig. 5, even at lower ion concentrations, ions (NH_4_^+^, Mg^2+^, NO_3_^−^, and PO_4_^3−^) were adsorbed to the biofilm and ion-exchange resins, and then the adsorption leveled off at higher ion concentrations; however, ions were not adsorbed to electrically neutral resin (data not shown). The adsorption of ions, both to biofilm and ion-exchange resins, could be fitted well by using the Langmuir isotherm model (*r*^2^≥0.95). The maximum adsorption amount (*N*_max_) and the adsorption equilibrium constant (*b*) values of the ion adsorption were calculated using a variant of the Langmuir isotherm model (3, 10, 19, 28) (Table 2), as described in the Materials and Methods section.

*N*_max_ for NH_4_^+^ to acidic resins (0.98 mmol dry-g^−1^ and 0.75 mmol dry-g^−1^ for strongly acidic and weakly acidic resins, respectively) was about twice that for Mg^2+^ (0.48 mmol dry-g^−1^ and 0.30 mmol dry-g^−1^ for strongly acidic and weakly acidic resins, respectively), and *N*_max_ for NO_3_^−^ to basic resins (1.7 mmol dry-g^−1^ and 0.63 mmol dry-g^−1^ for strongly basic and weakly basic resins, respectively) was approximately 3 times that for PO_4_^3−^ (0.50 mmol dry-g^−1^ and 0.20 mmol dry-g^−1^ for strongly basic and weakly basic resins, respectively).

Each adsorption site on the ion-exchange resins has the same affinity for a counter ion. In order to maintain electroneutrality, these adsorption sites are balanced by an equal amount of charge on the adsorbed counter ion. The higher the valency of the adsorbing ion, the greater the amount of opposite charge at the adsorption site on the ion-exchange resin required to adsorb that ion (5, 30). Hence, for the same amount of ion-exchange resin, *N*_max_ for divalent and trivalent ions is approximately a half and one-third, respectively, of *N*_max_ for monovalent ions.

However, for the biofilm, *N*_max_ for Mg^2+^ (1.3 mmol dry-g^−1^) was greater than that for NH_4_^+^ (1.1 mmol dry-g^−1^), and *N*_max_ for NO_3_^−^ (mmol dry-g^−1^) was twice that for PO_4_^3−^ (0.28 mmol dry-g^−1^) (Table 2). This result indicates that for the same amount of biofilm, *N*_max_ for the divalent cation is greater than that for the monovalent cation, and *N*_max_ for the trivalent anion is half that of the monovalent anion. It seems that in contrast to the ion-exchange resins, ion adsorption to the biofilm may not only occur by the ion-exchange mechanism. The attractive electrostatic interaction between charges in the biofilm polymer and ions in the surrounding water may also be involved in ion adsorption to the biofilm, as described in the kinetics section. In this case, *N*_max_ values for the biofilm represent the total adsorption amount promoted by both attractive electrostatic interactions and the ion-exchange mechanism.

*N*_max_ for the cation per unit dry weight of biofilm was greater than that for the acidic resins, particularly for Mg^2+^ (Table 2); however, *N*_max_ for anions to the biofilm was smaller than that for the strongly basic resin and about the same as that for the weakly basic resin. These results indicate that for the same amount of dry weight, the biofilm has higher adsorption capacity for cations than acidic resins, less adsorption capacity for anions than strongly basic resins, and similar adsorption capacity for anions as weakly basic resins.

The biofilm showed a greater *N*_max_ value for cation adsorption than for anion adsorption. Thus, the biofilm seems to have higher adsorption capacity for cations than for anions. Although the separate contributions of the attractive electrostatic interaction and the ion-exchange mechanism to *N*_max_ cannot be estimated under these experimental conditions, one possible reason for the higher adsorption capacity of the biofilm for cations than for anions is that the biofilm seems to provide a greater attractive force and have more adsorption sites for cations than for anions. This corresponds well with the result of the EPM measurement, revealing that the biofilm has many more negatively charged sites than positively charged sites at pH 7.

The values of *b*, which corresponds to the ratio of adsorption and desorption rates for each ion, were also calculated. The value of *b* in the case of ion-exchange resins represents the ratio of adsorption and desorption rates in the ion-exchange mechanism; however, the value of *b* for the biofilm represents the ratio of adsorption and desorption rates between ions in the surrounding water and in the biofilm matrix, where ion adsorption is promoted by both the attractive electrostatic interaction and the ion-exchange mechanism.

For all the ions considered, the value of *b* for the biofilm was smaller than that for the ion-exchange resins (acidic and basic resins). This indicates that ions are more easily desorbed from the biofilm matrix than from ion-exchange resins. Although we cannot know the relative contributions of the attractive electrostatic interaction and the ion-exchange mechanism to the desorption process, the lower *b* value for the biofilm may indicate that ions are more loosely bound to and more easily desorbed from the biofilm polymer. In this case, microbes would be able to use these ions as nutrients more efficiently. This result suggests that loosely binding ions and ion desorption from the biofilm polymer, after ion adsorption from the surrounding environment, may have an important role in providing nutrient ions to the microbes in the biofilm.

In the adsorption of ions (cations and anions) both to the biofilm and the ion-exchange resins, ions with a higher valency showed a greater value of *b* than those with a lower valency. The greater attractive force between the ions and the biofilm or resins seems to result in a greater ability to retain ions. This finding corresponds well with the results of the ion adsorption kinetics, which revealed that the divalent cations seem to be retained more strongly in the biofilm matrix than the monovalent cations.

There is a possibility that lysis of bacterial cells or surrounding extracellular polymeric substances (EPS) also affected the value of *b* under these experimental conditions. In order to assess this possibility, control experiments (using a biofilm suspension treated with the same procedure without ion addition) were conducted. The results showed no change in the ion concentrations of the solution from the beginning until the end of the experiment (60 min) (data not shown). This indicates that lysis of bacterial cells or EPS seems to have no effect on the value of *b* under these experimental conditions.

Although the *b* value (adsorption rate/desorption rate) has been investigated in other studies, they have been limited in two respects: 1) only heavy-metal ions adsorbing to 2) bacterial cell surfaces, bacterial biomass, or biofilms formed under artificial conditions have been studied (1, 6, 8, 9, 12, 14, 15, 21–25). To the best of our knowledge, the present study is the first to consider the *b* value of various ions, including nutrient ions, for biofilms formed in a natural environment.

There are some concerns that not only nutrient ions but also ions harmful to microbes may be adsorbed via ion-adsorption mechanisms and deposited in biofilms in the environment. This may be considered a disadvantage of the ion-adsorption mechanism for microbes in the biofilm. Further study of the adsorption and desorption of various ions, including ions harmful to microbes, to and from biofilms formed in natural environments, is necessary to clarify the advantages and disadvantages of ion adsorption for microbes in biofilms. However, from an environmental biotechnology point of view, the adsorption of harmful ions, such heavy metals, to biofilms may be applied to methods for the purification of pollutants in the environment, as reported in other studies (11, 19, 28, 29).

The present study demonstrated the following: 1) functional groups such as carboxylic and amino groups exist in the biofilm polymer; 2) the ionization of these functional groups causes biofilms to carry both positive and negative charges in the natural environment; 3) the physicochemical interaction between charges in the biofilm polymer and ions in the surrounding water seems to promote ion adsorption to the biofilm through an attractive electrostatic interaction and an ion-exchange mechanism; and 4) ions are loosely bound to and easily desorbed from the biofilm polymer after adsorption from the surrounding environment, and hence, microbes may readily use these ions as nutrients. To the best of our knowledge, this is the first study in which the equilibrium constant of ion adsorption to biofilms formed in a natural environment was investigated, and it was revealed that the ion adsorption-desorption characteristics of biofilms may have an important role in the supply of nutrient ions to microbes in biofilms and in the development of a nutrient-rich environment within biofilms.

## Figures and Tables

**Table 1:** Adsorbed and desorbed amount of ions to and from biofilm and ion exchange resins

Adsorbent	Adsorbed amount (mmol dry-g^−1^)		Desorbed amount (mmol dry-g^−1^)					
	
	Ion	amount	NH_4_^+^	K^+^	Ca^2+^	Mg^2+^	Na^+^	Total
Biofilm	NH_4_^+^	0.103	—1	0.0011	0.0091	0.0031	0.026	0.039
	K^+^	0.14	0.0021	—1	0.031	0.007	0.035	0.075
	Ca^2+^	0.205	0^3^	0.0011	—1	0.014	0.031	0.046
	Mg^2+^	0.25	0.0021	0.0051	0.039	—1	0.042	0.088

Strongly acidic resin	NH_4_^+^	0.059	—2	—2	—2	—2	0.041	0.041
	K^+^	0.063	—2	—2	—2	—2	0.078	0.078
	Ca^2+^	0.1	—2	—2	—2	—2	0.2	0.2
	Mg^2+^	0.095	—2	—2	—2	—2	0.2	0.2

Weakly acidic resin	NH_4_^+^	0.045	—2	—2	—2	—2	0.055	0.055
	K^+^	0.031	—2	—2	—2	—2	0.038	0.038
	Ca^2+^	0.065	—2	—2	—2	—2	0.11	0.11
	Mg^2+^	0.05	—2	—2	—2	—2	0.12	0.12

1 Examined adsorbed ions.

2 Not found in ion exchange resin (ion exchange resins only have Na^+^ as previously attached exchangeable ions).

3 Under detection limit.

**Table 2:** Maximum adsorption amount (*N*_max_) and adsorption equilibrium constant (*b*) of various ions, calculated using Langmuir isotherm model, to biofilm and ion exchange resins

Adsorbent	Adsorbate	*N*_max_ (mmol dry-g^−1^)	*b* (L mmol^−1^)
BF	NH_4_^+^	1.1±0.2*	0.040±0.012*
	Mg^2+^	1.3±0.1*	0.091±0.008*
	NO_3_^−^	0.57**	0.085**
	PO_4_^3−^	0.28**	0.11**

Strongly acidic resin	NH_4_^+^	0.98±0.17*	0.10±0.04*
	Mg^2+^	0.48±0.02*	1.3±0.6*

Weakly acidic resin	NH_4_^+^	0.75±0.05*	0.10±0.01*
	Mg^2+^	0.30±0.02*	1.3±0.6*

Strongly basic resin	NO_3_^−^	1.7±0.01*	0.57±0.01*
	PO_4_^3−^	0.50±0.02*	1.4±0.01*

Weakly basic resin	NO_3_^−^	0.63±0.01*	0.85±0.01*
	PO_4_^3−^	0.20±0.01*	1.4±0.1*

* Experiments were repeated 2–3 times, independently. ± values represent the standard error.

** Due to the limitation of the sample, the experiment was conducted once.
